# Large Subependymoma Inferior to the Cerebellopontine Angle With Significant Obstructive Hydrocephalus: A Case Report on an Extremely Rare Tumor

**DOI:** 10.7759/cureus.18686

**Published:** 2021-10-11

**Authors:** Pedram Laghaei Farimani, Mostafa Fatehi, Bradley M Chaharyn, Ryojo Akagami

**Affiliations:** 1 Division of Neurosurgery, Vancouver General Hospital, Vancouver, CAN; 2 Department of Medicine, University of British Columbia, Vancouver, CAN; 3 Department of Clinical Neurosciences, University of Calgary, Calgary, CAN; 4 Division of Neuropathology, Vancouver General Hospital, Vancouver, CAN

**Keywords:** subependymoma, cerebellopontine angle, benign brain tumor, rare brain tumors, rare case report, pontocerebellar cistern

## Abstract

Subependymomas are rare yet benign tumors that are commonly found within the ventricular system. We describe the case of a 51-year-old male presenting with hydrocephalus and progressive headaches found to have a right cerebellopontine angle (CPA) lesion encasing multiple blood vessels and cranial nerves (CN). The lesion was resected subtotally via a retrosigmoid approach and was found to be a subependymoma. CPA subependymomas are extremely rare lesions. The neuroimaging and histopathological findings as well as a comprehensive literature review of similar cases are discussed.

## Introduction

Intracranial subependymomas are rare, slow-growing tumors that are typically found in the fourth ventricle, followed by the lateral ventricles [1]. Many are discovered incidentally or during autopsy in middle-aged and older adults, more frequently in men, while symptomatic subependymomas commonly present with hydrocephalic symptoms or local mass effects [2,3]. Histologically, they appear benign and are categorized under WHO grade I neoplasms [2]. Furthermore, they have been found to account for 0.2%-0.7% of all intracranial tumors, with an older study finding an incidence of 0.57% and a more recent study finding 0.51% [4-6].

Microscopically, subependymomas consist of clusters of nuclei with coarse fibrillar matrices and lobular architecture, with microcysts being a defining MR feature [4,7]. In some cases, calcifications or intratumoral bleeding have been observed [5,8-10]. In preoperative scans, subependymomas were shown to be hypo- to isointense in T1-weighted MRI scans and hyperintense in T2-weighted MRI scans with respect to brain parenchyma [3].

A review of the literature suggests that extraventricular subependymomas are extremely rare [11]. Here, we present the case of a 51-year-old male who presented with symptoms of hydrocephalus and progressive headaches and was found to have a right cerebellopontine angle (CPA) lesion encasing multiple cranial nerves (CN) and blood vessels. The lesion was resected and was found to be a subependymoma. A comprehensive review of the literature found four similar reports. In addition to discussing the clinical features and pathology of the present case, we describe the relevant characteristics of other CPA subependymomas.

## Case presentation

Clinical history and examination

**Figure 1:**
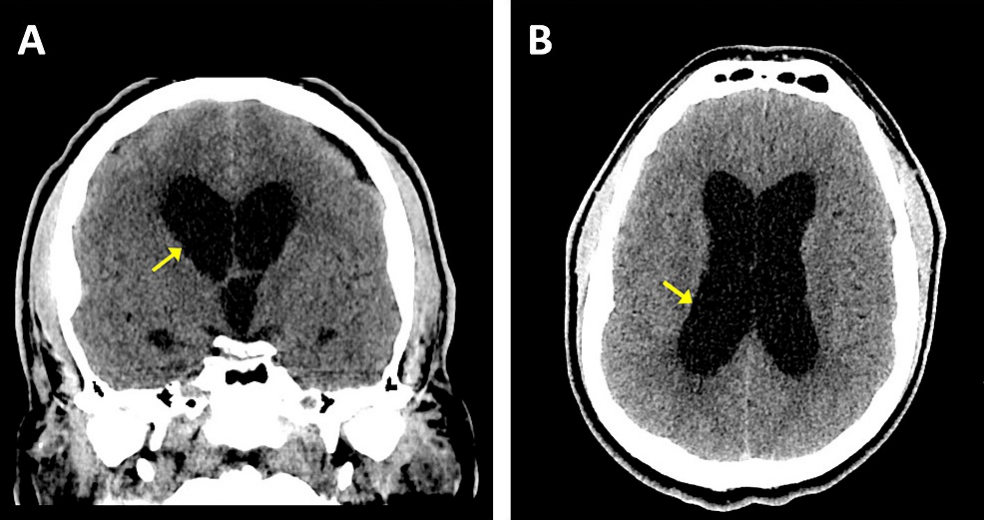
Preoperative CT findings revealing significant obstructive hydrocephalus shown by arrows in the coronal plane (A) and axial plane (B).

**Figure 2:**
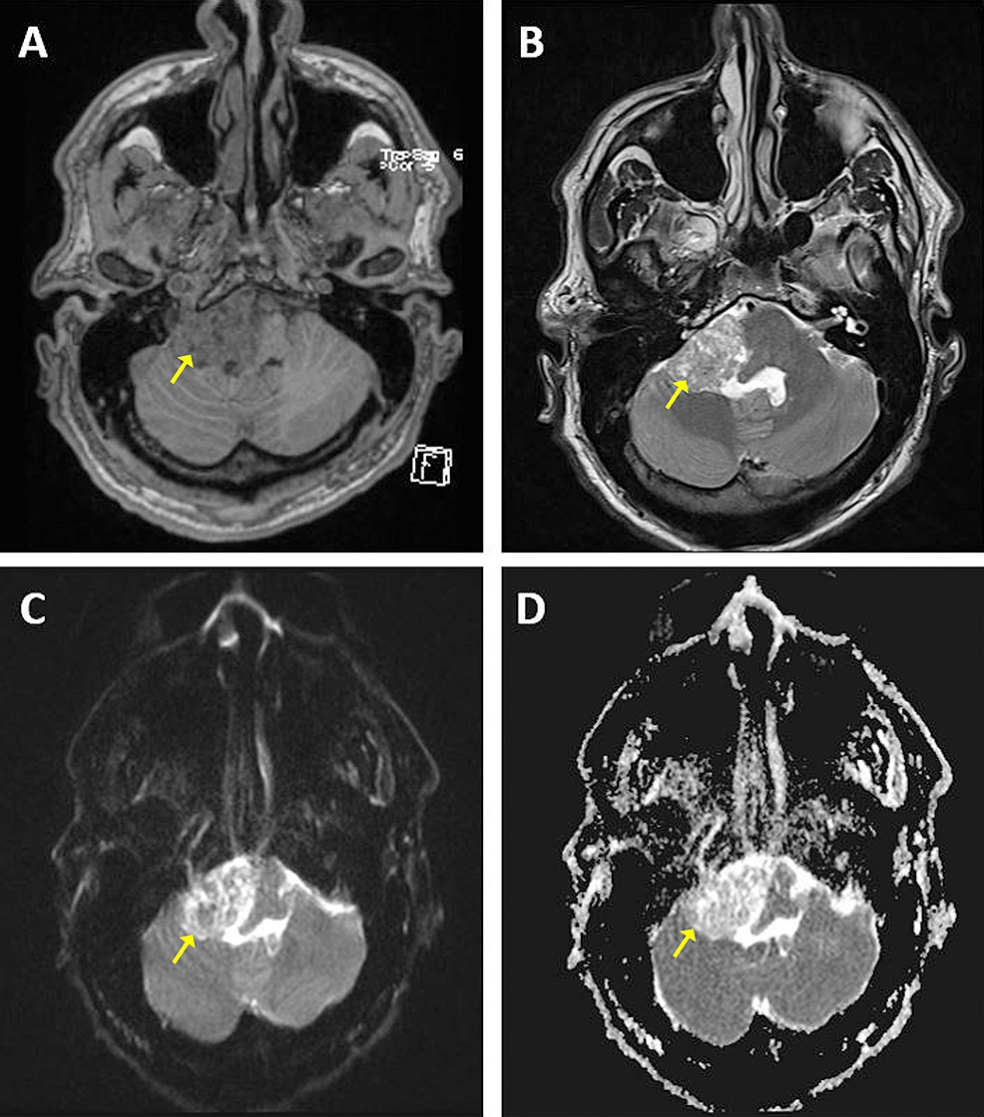
Preoperative MRI findings portraying roughly the same axial plane at varied sequences: T1-weighted image (A), T2-weighted image (B) illustrating a large hypodense and hyperdense right CPA mass shown by arrows, diffusion-weighted imaging (C), and apparent diffusion coefficient imaging (D).

A 51-year-old male presented with a history of several months of progressively worsening headaches, visual disturbances, and gait imbalance. Physical examination revealed papilledema and poor tandem gait. A CT scan was obtained (Figure 1), which showed obstructive hydrocephalus, and an MRI scan (Figure 2), which showed a large right CPA mass. The imaging findings were most consistent with a glomus jugulare or vestibular schwannoma with significant compression of the adjacent medulla and pons and obstructive hydrocephalus with a patent aqueduct. A right retrosigmoid approach was performed with intraoperative monitoring for resecting the tumor, namely, intraoperative neurophysiological monitoring with cranial nerve (CN) V, VII, X, and XII EMG monitoring, CN VII and X motor evoked potentials, bilateral brainstem auditory evoked responses, four-limb somatosensory evoked potentials, and motor evoked potentials. Intraoperatively, some tumor tissue was sent for frozen section pathology, which suggested some ependymal-type cells not consistent with a schwannoma or a meningioma. As the tumor was found to encase multiple nerves, including CN VII, X, and XII, a decision was made to only attempt subtotal resection. The intraoperative monitoring did not suggest any significant changes, and the patient was intact.

Neuroimaging findings

**Figure 3:**
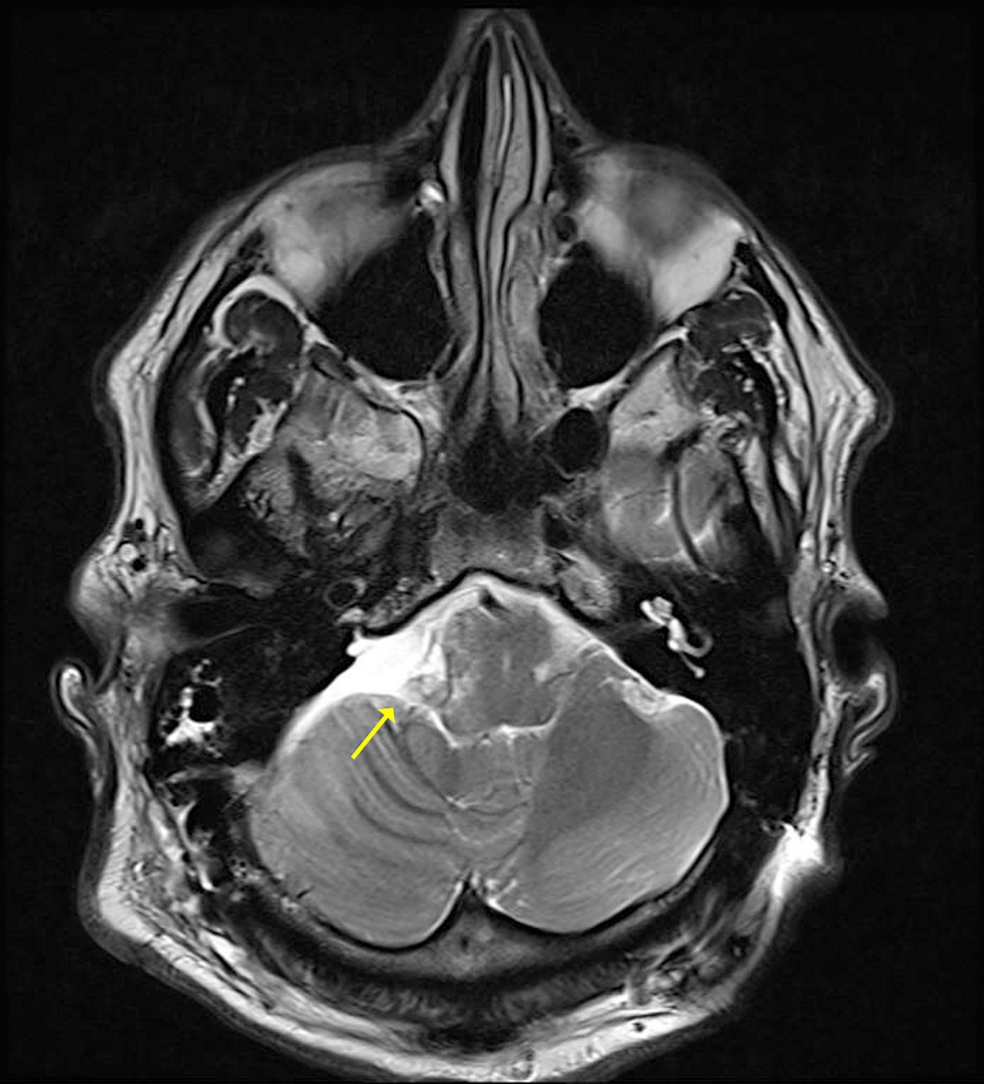
A postoperative T2-weighted image following a subtotal resection of the tumor shown by the arrow.

As previously discussed, preoperatively, a coronal and axial CT were obtained, which revealed obstructive hydrocephalus in the lateral ventricles (Figure 1). Brain MRI was followed and revealed a large mass located in the posterior fossa, inferior to the right CPA, along with a collection of extra-axial fluid. The lesion appeared to be hypodense in T1-weighted MRI (Figure 2A) and hyperdense in T2-weighted MRI (Figure 2B). Although imaging revealed severe compression of the adjacent medulla and pons in addition to partially encasing the right vertebral artery, the cerebral aqueduct was found to be patent. Furthermore, neuroimaging findings were most consistent with a glomus jugulare or vestibular schwannoma. MRI was obtained after subtotal resection (Figure 3).

Surgical procedure

The operation was performed via a right retrosigmoid approach. During debulking, it was noted that the tumor had a slightly unusual consistency, not typical for a schwannoma. A quick frozen section pathology was requested and revealed some ependymal-type cells not consistent with a schwannoma or a meningioma. After further debulking, multiple blood vessels were seen to be exiting the tumor, which was also quite unusual. Moreover, the tumor was found to encase CN VII, X, and XII. Due to this anatomy, a decision was made to perform a subtotal resection; the tumor between the vertebral artery, basilar artery, posterior inferior cerebellar artery, and brainstem, where perforators would be penetrating the tumor to get to the brainstem, was left behind.

CN VII motor evoked potentials changed slightly in morphology but maintained good amplitudes. The brainstem auditory evoked responses decreased during the procedure but recovered close to baseline by the end. The monitoring for the other nerves and brainstem remained unchanged throughout the procedure.

Histopathological findings

**Figure 4:**
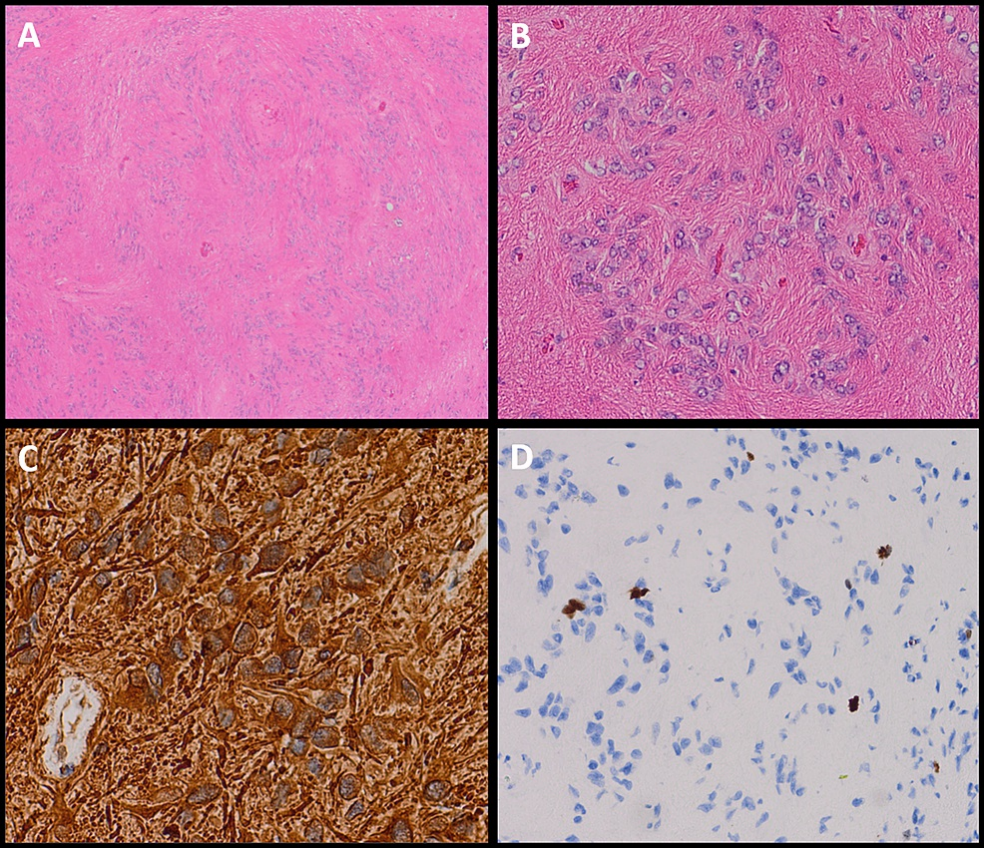
Microscopic sections of the tumor reveal small uniform cells in clusters embedded in a dense fibrillar background (A, B). The tumor cells are strongly immunoreactive for GFAP (C) and have a Ki67 proliferative index of less than 1% (D). H&E 4x (A) and 20x (B); GFAP 40x (C); and Ki67 20x (D).

The tumor was received fresh from the operating room and subsequently fixed in formalin and embedded in paraffin. Routine hematoxylin and eosin (H&E)-stained sections revealed classic features of a subependymoma. The sections show a paucicellular tumor composed of small clusters of glial cells embedded in a dense fibrillar network (Figure 4). The tumor nuclei are uniformly small and round with fine dispersed chromatin and no discernable mitotic activity. Necrosis and microvascular proliferation are absent. Immunohistochemistry reveals strong cytoplasmic GFAP immunoreactivity and a Ki67 proliferation index of less than 1%.

Methods

**Figure 5:**
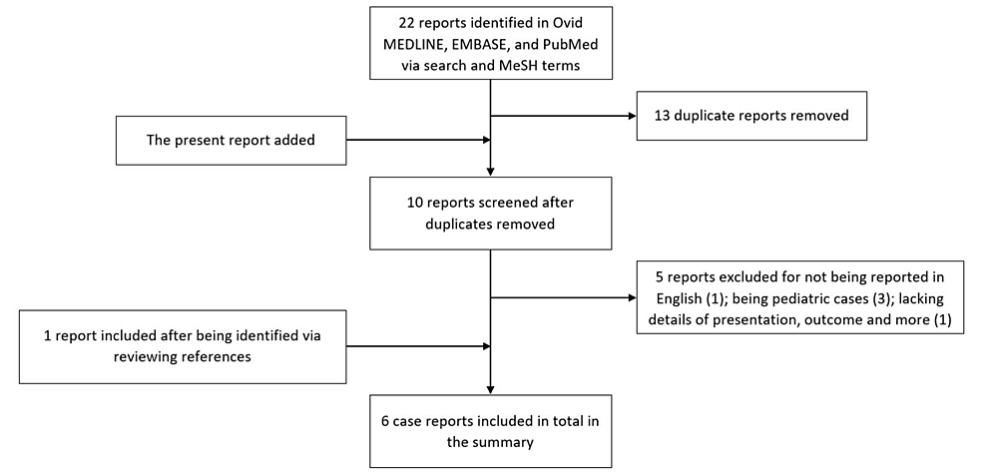
A PRISMA flow diagram showing the progression of the present study in identifying case reports of CPA subependymomas in adults.

A thorough search of the literature available on PubMed, Ovid MEDLINE, and EMBASE databases was conducted for case reports of CPA subependymomas. This was completed by including a combination of the following MeSH terms: “Brain Neoplasms/[Diagnosis, Surgery],” “Cerebellopontine Angle/[Pathology, Surgery],” “Glioma, Subependymal/[Diagnosis, Surgery],” and “Neuroma, Acoustic/[Diagnosis, Surgery].” The search resulted in a total of 22 case reports. After removing duplicates and several other reports for either not being reported in English, being pediatric cases, or lacking information, four case reports of CPA subependymomas were reviewed and summarized in Table 1, along with the present case; one of the reports included was identified via reviewing references (Figure 5).

## Discussion

Subependymomas are rare, benign tumors that are typically detected at 39-59 years of age in adults and males in 72% [8,10,12]. Due to their indolent growth rate, the likelihood that they remain asymptomatic throughout life is significant [6]. They frequently develop in the fourth ventricle (50%-60% of cases); followed by the lateral ventricles (30%-40% of cases); and, to a lesser extent, the third ventricle, spinal cord, and septum pellucidum [13]. It is, thus, extremely rare for subependymomas to develop in extraventricular sites, particularly when they are confined within the CPA [6,12,14]. Nevertheless, they should not be excluded as a differential diagnosis when a neoplasm is lacking ventricular involvement [15].

After a thorough review of the literature found on PubMed, Ovid MEDLINE, and EMBASE, four adult case reports were included and summarized, including the one we presently report, a 51-year-old male with a significant right low CPA subependymoma (Table 1). Cunha et al. published a case of a 57-year-old male with a large left CPA subependymoma, which expanded into the jugular foramen [13]. Hoeffel et al. presented eight cases, one of which described a case of a 52-year-old male with a subependymoma that developed in the left CPA and extended to the foramen of Luschka [9]. It was noted that this case was also the only one causing edema. Huang published their case of a 44-year-old female with a CPA subependymoma that compressed the brainstem and fourth ventricle [15]. Jooma et al. presented one case of a 47-year-old patient with a left CPA subependymoma out of 12 cases in their surgical series [10]. Finally, Matsumara et al. described one case of a 55-year-old male out of seven symptomatic subependymomas that was found attached to the floor of the fourth ventricle and extending to the left CPA [5].

**Table 1:** Summary of the demographics, neuroimaging findings, presentation, surgical procedure, and outcome of published cases of CPA subependymoma, including the one presented in the current case report. CPA, cerebellopontine angle; F, female; M, male; NR, not reported; V4, fourth ventricle; –, hypointense; +, hyperintense

Case	Publication	Age	Sex	Location	T1 Imaging	T2 Imaging	Presentation	Procedure	Outcome
1	Cunha et al. [13]	57	M	Left CPA	–	+	Daily occipital headache, non-vertiginous dizziness, nausea, gait disorder	Complete resection	Improved
2	Hoeffel et al. [9]	52	M	Left CPA	NR	+	NR	Surgery, but no further data available	NR
3	Huang [15]	44	F	CPA	–	+	Headache	Complete resection	Improved
4	Jooma et al. [10]	47	NR	Left CPA	NR	NR	NR	NR	Disabled, but good recovery after three years
5	Matsumara et al. [5]	55	M	Left CPA and V4	NR	NR	Three-year history of dizziness, blurred vision, mental disturbance, ataxia, nystagmus, tetraparesis, incontinence	Subtotal resection	Worsened
6	Present case	51	M	Right CPA	–	+	Worsening headaches, visual disturbance, gait disorder	Retrosigmoid, subtotal resection	Improved with stable, small residual

According to the literature, the histogenesis of subependymomas remains unclear. Different authors have proposed their origin to be from the development of subependymal glial precursor cells, astrocytes of the subependymal plate, ependymal cells, or a mixed type of astrocytic and ependymal cells [8,10,12]. This uncertainty is partly due to the varying degrees of astrocytic and ependymal cells in mixed areas within these tumors [16]. A relatively recent theory proposes that they originate from tanycyte cells, which are typically found in the subependymal zone; ultrastructurally, tanycytes show features resembling astrocytic and ependymal cells similar to the presentation of subependymomas [17].

Surgical treatment is typically indicated for cases of symptomatic subependymomas. In fact, complete resection, when possible, is often curative and favored as subependymomas are benign, indolent, and non-infiltrative [6]. However, treatment should prioritize safely resecting the tumor, decompressing neural elements, establishing a pathological diagnosis, and restoring normal pathways for cerebrospinal fluid [13]. Furthermore, it appears that the relationship between the extent of resection and survival rates is insignificant; age at the time of surgery seems to be the only variable that significantly affects survival, with patients over 50 years of age having a worse prognosis [11]. Although the role of radiotherapy is unclear, adjuvant radiation therapy has been proposed for the subsequent management of subtotally resected or recurrent subependymomas, but no consensus has been reached in the academic community [18].

## Conclusions

In conclusion, we report a relatively rare case of a right CPA subependymoma encasing multiple cranial nerves and blood vessels that was resected subtotally via a right retrosigmoid approach. Although very unlikely, a lesion exclusive to the CPA without ventricular involvement should not exclude subependymoma from the differential diagnosis. In fact, it is vital for the purposes of planning the operation to have a preoperative suspicion of subependymoma. Furthermore, to resect the mass in a subtotal fashion may be an appropriate choice, as prognosis seems to be independent of the extent of resection.
